# Discovery of a Remarkable Methyl Shift Effect in the Vanilloid Activity of Triterpene Amides

**DOI:** 10.1021/acs.jnatprod.0c00639

**Published:** 2020-11-02

**Authors:** Rosa Maria Vitale, Cristina Avonto, Danilo Del Prete, Aniello Schiano Moriello, Pietro Amodeo, Giovanni Appendino, Luciano De Petrocellis

**Affiliations:** † Institute of Biomolecular Chemistry, National Research Council (ICB-CNR), Via Campi Flegrei 34, 80078 Pozzuoli (NA), Italy; ‡ National Center for Natural Products Research, Research Institute of Pharmaceutical Science, School of Pharmacy, The University of Mississippi, University, Mississippi 38677, United States; § Dipartimento di Scienze del Farmaco, Università del Piemonte Orientale, Largo Donegani 2, 28100 Novara, Italy; ^ Endocannabinoid Research Group (ERG), Institute of Biomolecular Chemistry, National Research Council (ICB-CNR), Via Campi Flegrei 34, 80078 Pozzuoli (NA), Italy; ∥ Epitech Group SpA, Saccolongo, Padova, Italy

## Abstract

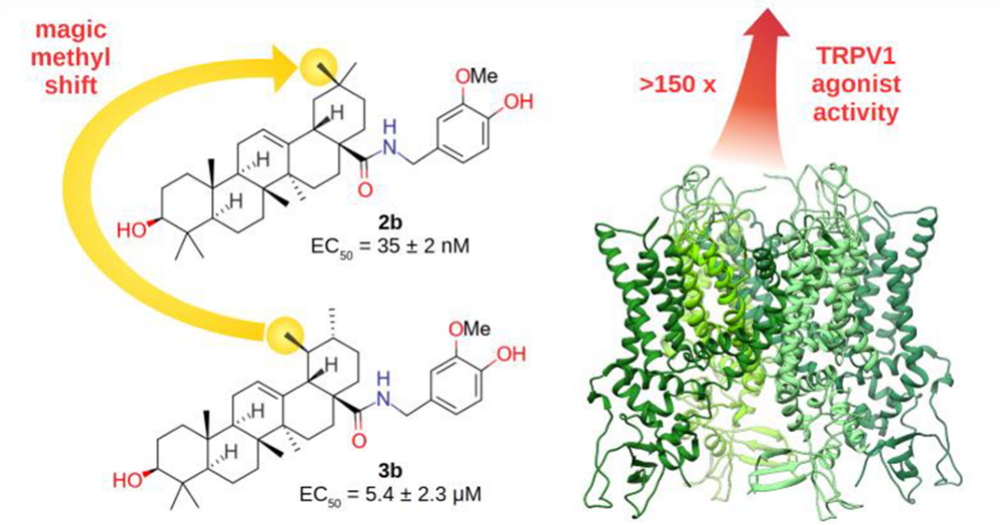

As part of a study on triterpenoid conjugates, the dietary pentacyclic triterpenoids oleanolic (**2a**) and ursolic acids (**3a**) were coupled with vanillamine, and the resulting amides (**2b** and **3b**, respectively) were assayed for activity on the vanilloid receptor TRPV1. Despite a structural difference limited to the location of a methyl group in their conformationally rigid pentacyclic core, oleanoloyl vanillamide dramatically outperformed ursoloyl vanillamide in terms of potency (EC_50_ = 35 ± 2 nM for **2b** and 5.4 ± 2.3 μM for **3b**). Using molecular docking and dynamics, this difference was translated into distinct accommodation modes at the TRPV1 vanillyl ligand pocket, suggesting a critical role of a C–H π^phenyl^ interaction between the triterpenoid C-29 methyl and Phe591 of TRPV1. Because the molecular mechanisms underlying the activation process of transient receptor channels (TRPs) remain to be fully elucidated, the observation of spatially restricted structure–activity information is of significant relevance to identify the molecular detail of TRPV1 ligand gating.

TRPV1, a member of the vanilloid subfamily of transient potential receptor channels (TRPs), is critically involved in the transduction of nociceptive stimuli and is responsible for the irritant and burning sensation of capsaicin (**1**), the active ingredient of hot chili pepper.^1^ Structure–activity studies have identified the critical structural determinants for TRPV1 activation by capsaicinoids,^2^ and cryo-EM studies of this ion channel bound to a set of ligands (the plant diterpenoid resiniferatoxin, the tarantula toxin DkTx, the synthetic antagonist capsazepine) have provided insights into protein–ligand interactions in the TRPV1 vanillyl pocket.^3−5^ However, given the complexity and multimodal action of this class of receptors, the molecular details of the ligand–channel interactions have largely remained elusive, in particular, and paradoxically, for the archetypal ligand capsaicin (**1**).^6^ To this aim, data that translate into unambiguous and spatially restricted structure–activity information are of considerable relevance. In this context, we report the discovery of a “magic” methyl shift effect^7,8^ in the activity of the vanillamides of oleanolic (**2a**) and ursolic acid (**3a**) and its rationalization in terms of docking and molecular dynamics (MD) experiments in the TRPV1 ligand pocket.

Oleanolic and ursolic acids (**2a** and **3a**, respectively) are pentacyclic triterpenoids with a broad distribution in Nature and with a significant human dietary exposure due to their occurrence in edible plants (olives), herbs (sage), fruits (apple),^9^ and caffeinated spices (mate).^10^ Their profile of bioactivity is of considerable interest in the realm of inflammation, pain, and metabolic syndrome, but their insolubility and dismally low oral absorption have so far prevented pharmaceutical advancement.^9^ Derivatization of the carboxylate function, as in bardoxolone methyl,^11^ is known to improve the pharmacokinetic profile of oleanolic and ursolic acids,^11^ and provided a rationale for the synthesis of conjugates of the natural compounds. Biogenic amines seemed an attractive class of conjugation candidates, as lipophilic acids like fatty acids can be converted in vivo in amide conjugates.^12^ Since oleanolic and ursolic acid show an intrinsic modest antagonist activity on TRPV1,^13,14^ conjugation with vanillamines seemed, in the context of control of pain and inflammation, a rational strategy to improve both the pharmacodynamic and the pharmacokinetic properties of the natural products.

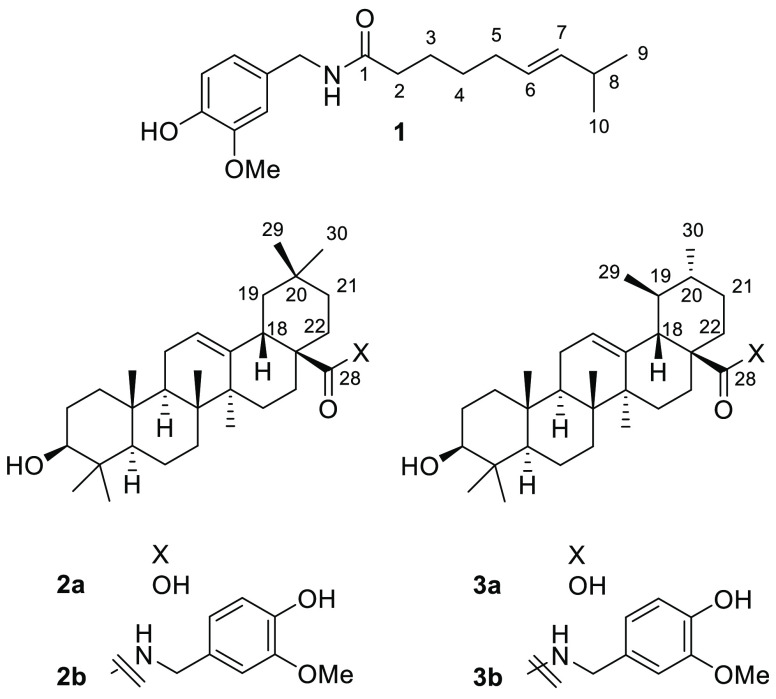



The neopentylic C-28 carboxylic group of triterpenoid acids is poorly reactive and requires forced conditions for its esterification and amidation, with additional complications observed for phenolic amines due to competition with the formation of phenolic esters.^15^ We previously developed a protocol for the amidation of triterpenoid acids with phenolic amines based on the activation of the carboxylic group by in situ formation of a mixed phosphoric anhydride.^15^ However, modest yields were obtained with ursolic acid, and yield further dropped with oleanolic acid, presumably because the *gem*-dimethyl substitution further encumbers the C-28 carboxylic group. Much better yields could, however, be obtained by ex situ activation with hydroxysuccinimide and reaction with an excess of free amine. In this way, improved yields (20–30%) were obtained for various amino alcohols and aminophenols, including vanillamine.

Vanilloid activity was evaluated in HEK-293 cells overexpressing hTRPV1. Despite the consistent tendency for compounds of the ursolic acid series to outperform those from the corresponding oleanolic series,^9^ about 2 orders of magnitude greater potency for the vanillamide of oleanolic acid was observed compared to that of ursolic acid (EC_50_ = 35 ± 2 nM for **2b** vs 5.4 ± 2.3 μM for **3b**, Table 1).

**Table 1:** TRPV1 Activity Data for the Vanillamides **2b** and **3b** Compared to the Activity of Capsaicin (**1**)

compound	efficacy (relative to ionomycin 4 μM)	potency EC_50_	IC_50_ (capsaicin 0.1 μM)
**2b** (oleanoyl vanillamide)	72 ± 1	35 ± 2 nM	50 ± 2 nM
**3b** (ursoloyl vanillamide)	16 ± 1	5.4 ± 2.3 μM	7.5 ± 0.7 μM
**1** (capsaicin)	79 ± 1	5.3 ± 0.4 nM	8.0 ± 0.3 nM

a Data were obtained in HEK-293 cells, stably transfected with recombinant human TRPV1 (hTRPV1).

The pentacyclic triterpenoid scaffold of oleanolic and ursolic acid is devoid of conformational mobility, and the two compounds only differ in the location of a methyl group, making it possible that the presence of a substitution at C-19 interferes, by steric hindrance, with the fitting of **3b** into the ligand binding site of TRPV1. However, a docking study by using the available structure of TRPV1 in its activated state (PDB id: 5IRX) suggested a more complex and different scenario. In fact, both compounds docked into the vanilloid-binding pocket, as defined by the S3–S4 helices, S4–S5 linker of one subunit, and the S5–S6 helices of the adjacent subunit.^10^ The two best not-redundant poses in terms of binding energy value for each compound (Figures 1 and 2, panel B) only differed in the orientation of the vanillyl moiety (hereinafter referred to as OMe-in when the methoxy group points toward the cleft between helices S3 and S4 and OMe-out when it is rotated by 180°).

**Figure 1:**
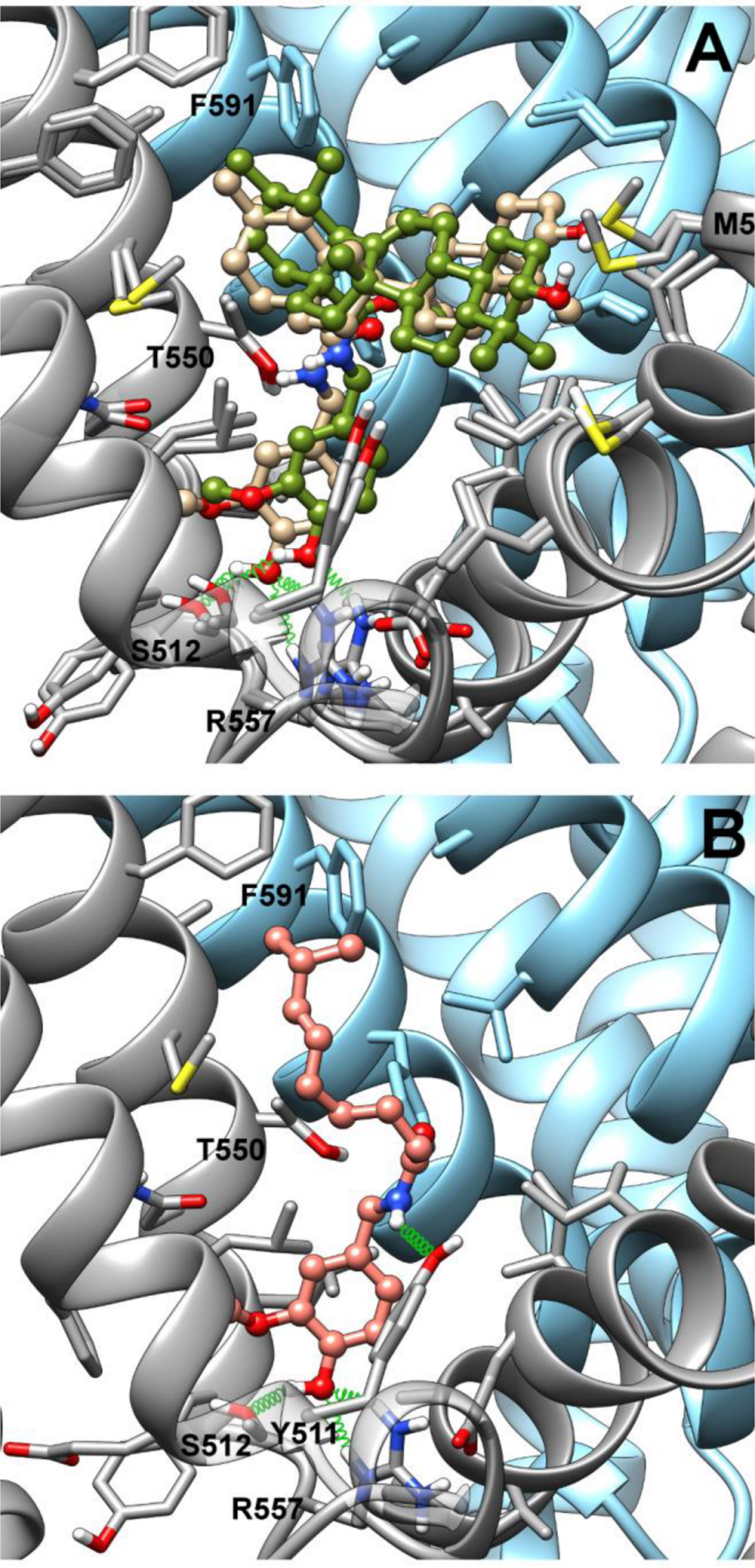
Representative energy-minimized OMe-in docking poses of **2b** and **3b** (tan and olive drab, respectively, panel A) and capsaicin (salmon, panel B) after best fit of the protein backbone. Ligands are shown in ball-and-stick representation, whereas protein residues within 4.5 Å from the ligand are shown in stick representation. Ribbons and selected side chain stick bonds of TRPV1 monomers A and B are colored in dark gray and sky blue, respectively. Oxygen, nitrogen, and sulfur atoms are colored in red, blue, and yellow, respectively. Only polar hydrogens are shown and colored white.

**Figure 2:**
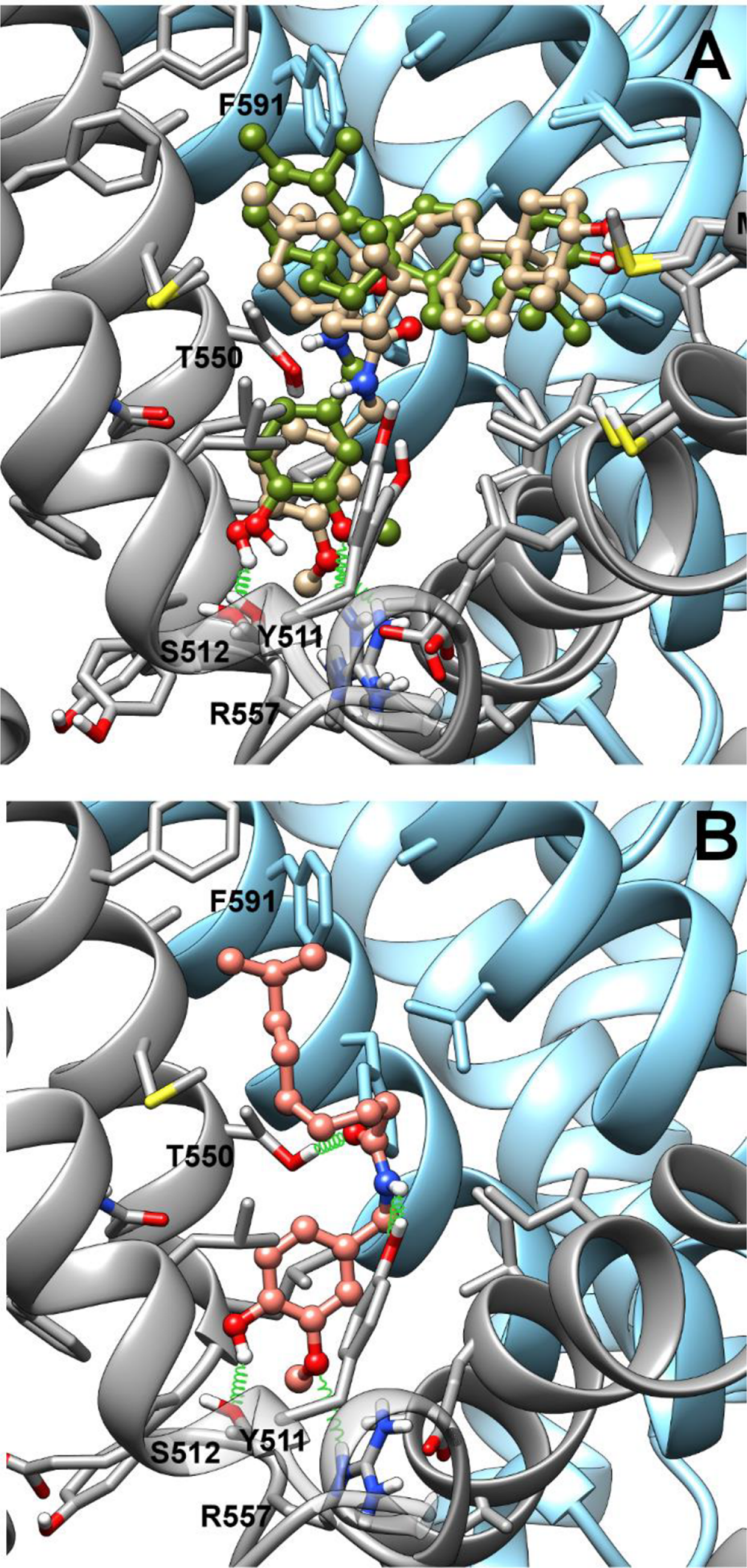
Representative energy-minimized OMe-out docking poses of **2b** and **3b** (tan and olive drab, respectively, panel A) and capsaicin (salmon, panel B) after best fit of the protein backbone. Ligands are shown in ball-and-stick representation, whereas protein residues within 4.5 Å from the ligand are shown in stick representation. Ribbons and selected side chain stick bonds of TRPV1 monomers A and B are colored in dark gray and sky blue, respectively. Oxygen, nitrogen, and sulfur atoms are colored in red, blue, and yellow, respectively. Only polar hydrogen are shown and colored white.

At odds with the starting hypothesis, in the emerging scenario the β-oriented methyl on C-19 does not prevent accommodation of **3b** in the binding site, but its translocation on C-20 rather induces a better fit of **2b** in the binding site. In fact, in both poses, the vanillyl group of **2b** is deeper inside the pocket than in **3b**, and this arrangement is promoted by a C–H π^phenyl^ interaction^16,17^ between the C-29 methyl group and Phe591 (S5 helix-B monomer) side chain, which pushes down the terpenoid scaffold. Conversely, the lack of this methyl in **3b** induces a shift of the terpenoid scaffold toward Phe591, resulting in a looser binding of the vanillyl group in the ligand pocket. In the OMe-in orientation, both vanillamides are engaged in H-bonds between the hydroxyl group and both the Ser512 (S3) and Arg557 (S4) side chains, whereas in the OMe-out orientation only the phenolic hydroxy of **2b** can form a H-bond with the Ser512 side chain, whereas the methoxy groups of both isomers are H-bonded to the Arg557 side chain. The C-3 hydroxy of the cyclic scaffold of both isomers is close to the sulfur atom of the Met581 (S4–S5 linker) side chain in both the OMe-in and OMe-out orientation. Because in rigid systems the effect of substitution can be directly translated into the occupancy of a specific area of the ligand-binding space, it was interesting to investigate if the site of the C–H π^phenyl^ interaction of the C-29 oleanoyl methyl was also occupied by capsaicin. When this archetypal vanilloid ligand was docked into the vanilloid-binding pocket, one of the ω-methyls was indeed spatially close to Phe591 (S5-B), with two orientations of the vanillyl group of the same OMe-in and OMe-out type being observed, in accordance to the binding mode of **2b** and **3b** (Figures 1 and 2, panel B). On account of a major conformational mobility and a slender carbon–carbon connectivity, the branched acyl tail of capsaicin allows two H-bonds of its amide group with either both the Thr550 (S4) and the Tyr511 (S3) side chains (OMe-out orientation) or, alternatively, Tyr511 (S3) (OMe-in orientation), rationalizing the higher potency of capsaicin compared to **2b** (EC_50_ = 5.3 and 35 nM, respectively). For comparison purposes, we also evaluated the activity of the corresponding acidic parent triterpenoids (oleanolic and ursolic acids, **2a** and **3a**, respectively), previously reported to act as weak antagonists at TRPV1.^13,14^ We confirmed that both compounds behave as weak antagonists, inhibiting the capsaicin response by 20 ± 3% and 30 ± 1% at 25 μM, respectively. The corresponding docking complexes are reported in Figure S1. Ursolic acid (**2a**) engages a H-bond between its carboxylate and the Thr550 side chain, while the arrangement of the polycyclic moiety is substantially preserved in comparison to its vanillamide-conjugated derivative. Conversely, oleanolic acid adopts a completely different orientation, engaging Ser512 with a H-bond with its hydroxy group. A hypothetical corresponding pose of ursolic acid, with the carboxylate group forming a H-bond with Thr550, is prevented by a steric clash between the C-29 methyl group and Phe591. Thus, since both acidic precursors are endowed with a weak and comparable inhibitory activity, the dramatic difference in the activity profile between **2b** and **3b** can be ascribed to the introduction of a vanillamide group. To confirm and further explore the better accommodation of **2b** vs **3b** within the site emerging from the docking, we carried out 100 ns of molecular dynamics in the membrane environment for both OMe-in complexes. The root mean square deviation (rmsd) of both protein and ligands, shown in Figure 3, shows smaller fluctuations in both protein and ligands for the **2b** complex in comparison with those of **3b**. In fact, the latter is characterized by both a drift in protein backbone and a higher mobility of the ligand in the four binding sites of the tetramer. Thus, MD calculations show a relative structural destabilization on going from **2b** to **3b** of the active form of TRPV1 used to derive the theoretical complexes, corresponding to the cryo-EM structure in complex with resiniferatoxin. The greater structural stability of **2b** is also confirmed by the network of H-bonds engaged within each binding site in comparison to that of **3b**, as shown in Table 2, reporting H-bond occurrences greater than 10% over the simulated 100 ns of production run. In fact, while **3b** forms only one H-bond with Ser152 with an occurrence of ∼40% in three sites out of four, **2b** forms additional H-bonds with Arg557 and/or Glu570, with an overall occurrence of H-bonds well above 50%, up to ∼74%. Moreover, methyl C29 forms stable hydrophobic interactions with both Phe591 and Ala549 during the whole simulated period, as shown in Figure S2. The representative frames from molecular dynamics are shown in Figure 4. The greater capability of **2b** to stabilize the active form of TRPV1 is fully consistent with the higher agonist efficacy observed for this compound in comparison with **3b**.

**Figure 3:**
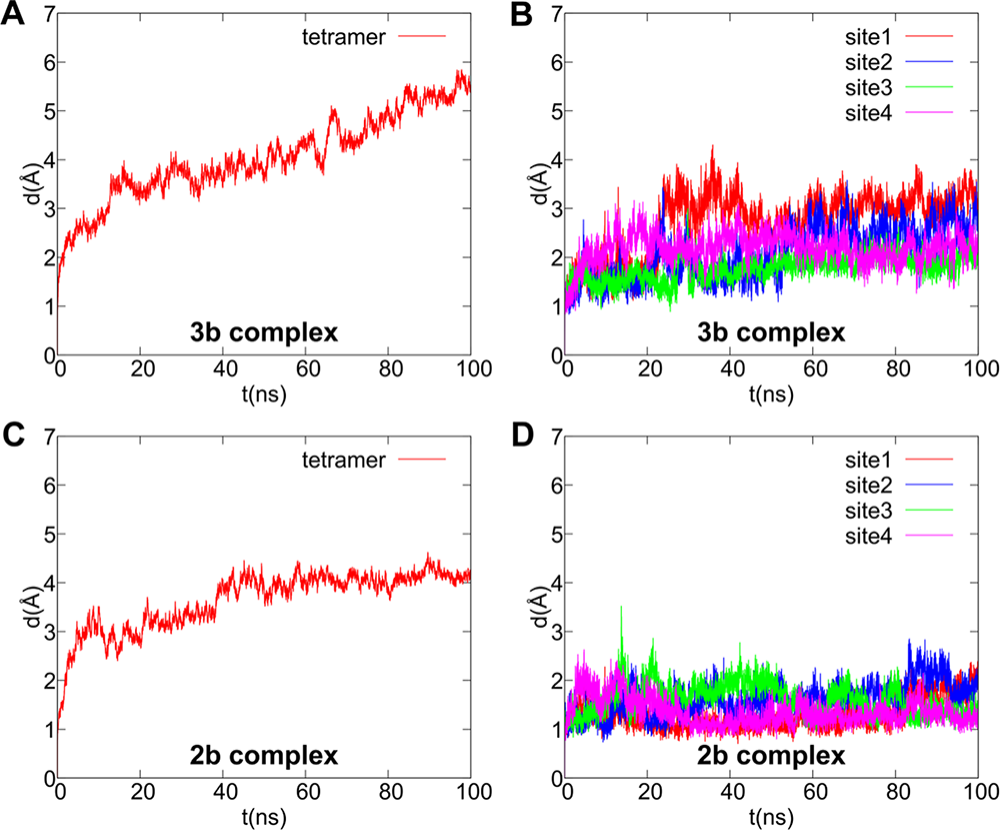
Root mean square deviation (rmsd) of protein backbone atoms (panels A, C) and the respective ligands (B, D) after protein best fit. Plot lines were smoothed with a five-point window running average.

**Table 2:** Occurrence of Ligand–Protein H-Bonds for TRPV1 in Complex with Compounds **3b** and **2b** during 100 ns of MD

protein binding site	compound **3b** (HB occurrence)	compound **3b** (frames with ≥1 HB)	compound **2b** (HB occurrence)	compound **2b** (frames with ≥1 HB)
1	OH···Ser512 (36.8%)	42.42%	OH···Ser512 (51.2%)	52.80%
2	OH···Ser512 (34.8%)	44.28%	OH···Ser512 (64.2%); OH···Glu570 (50%)	73.8%
3	OH···Ser512 (40.8%)	40.99%	OH···Ser512 (41.0%); OH···Arg557 (39.7%); CO···Tyr511 (33%)	66.80%
4	OH···Ser512 (58.7%)	58.7%	OH···Ser512 (57.6%)	57.6%

**Figure 4:**
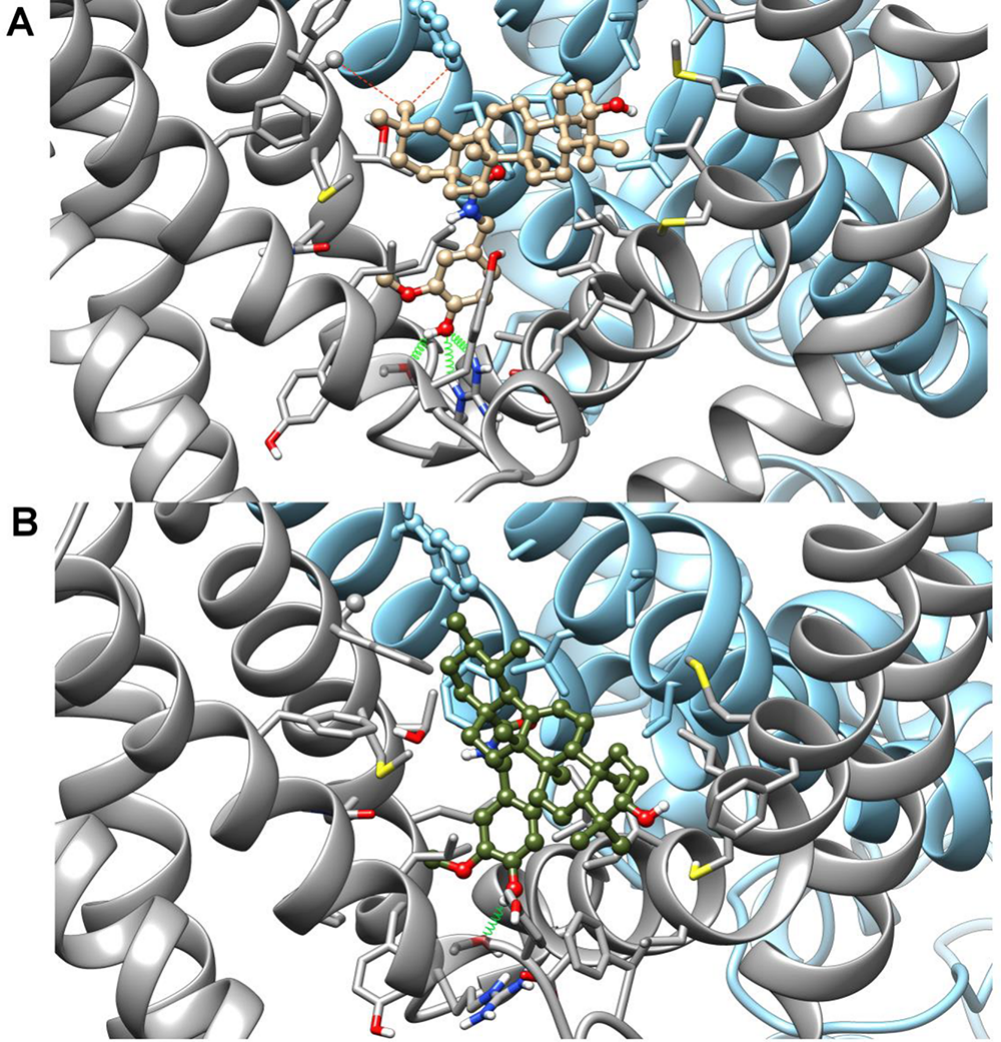
Representative frames from MD of **2b** (A) and **3b** (B) complexes with TRPV1. The color code is the same used for Figure 2. Red dotted lines represent the distance between C29 and both Ala549 and Phe591 in complex **2b**.

In conclusion, a comparative analysis of bioactivity data, docking experiments, and MD simulations has highlighted the critical role of the C-29 methyl of triterpenoids for significant and effective binding to TRPV1, with only the oleanane skeleton having this methyl in the correct location for the interaction. As the rigid ring system of both triterpenoid vanillamides encompasses conformationally constrained versions of the side chain of capsaicin, it is not unrealistic that a similar interaction may occur between Phe591 and one of the ω-methyls of capsaicin, thus disclosing a role for this residue in agonist binding and receptor activation.

## Experimental Section

### General Experimental Procedures

IR spectra were obtained on an Avatar 370 FT-IR Thermo Nicolet. ^1^H (300 MHz) and ^13^C (75 MHz) NMR spectra were measured on a Bruker spectrometer. ^1^H (500 MHz) and ^13^C (126 MHz) NMR spectra were measured on an Agilent spectrometer. Chemical shifts were referenced to the residual solvent signal (CDCl_3_, δ_H_ = 7.26, δ_C_ = 77.16, or DMSO-*d*_6_, δ_H_ = 2.50, δ_C_ = 39.52, hept). Low- and high-resolution ESIMS spectra were obtained on an LTQ OrbitrapXL (Thermo Scientific) mass spectrometer. Silica gel 60 (63–200 mesh) used for gravity column chromatography was purchased from Merck. Reactions were monitored by TLC on silica gel Merck 60 F254 (0,25 μm) plates and neutral alumina Macherey-Nagel ALUGRAM (0,20 μm) plates that were visualized by UV inspection (254 and 365 nm) and/or staining with 5% H_2_SO_4_ in EtOH and heating. Organic phases were dried with anhydrous Na_2_SO_4_ before evaporation. Chemical reagents and solvents were from Sigma-Aldrich.

### Synthesis of Triterpenoid Vanillamides. Synthesis of Oleanoyl Vanillamide (**2b**) as Representative

(a) Carboxylate activation: To a stirred solution of *N*-hydroxysuccinimide (1.51 g; 13.1 mmol) in EtOAc (50 mL) were added oleanolic acid (2.05 g; 4.5 mmol) and dicyclohexylcarbodiimde (DCC, 4.58 g; 22.2 mmol). The suspension was stirred at room temperature (rt) for 16 h and then worked up by filtration and evaporation. The residue was purified by gravity column chromatography using petroleum ether/EtOAc (8:2) as mobile phase, to give the hydroxysuccinimide ester as a white powder (1.32 g, 52% yield): ^1^H NMR (300 MHz, CDCl_3_) δ 5.31 (1H, brt), 3.46 (1H, m), 3.20 (1H, m), 2.79 (4H, m), 1.15 (3H, s), 0.98 (3H, s), 0.92 (3H, s), 0.91 (3H, s), 0.90 (3H, s), 0.80 (3H, s), 0.76 (3H, s). (b) Amidation: To a stirred solution of oleanoyl hydroxysuccinimide (300 mg, 0.54 mmol) in CH_2_Cl_2_ (4 mL) was added vanillamine (150 mg, 1.1 mmol). The mixture was stirred at rt for 24 h and then worked up by dilution with brine and extraction with CH_2_Cl_2_. The organic phase was treated with Na_2_SO_4_ and filtered, and the solvent evaporated. The residue was purified by gravity column chromatography using petroleum ether/EtOAc (3:7) to give **2b** as a white powder (66 mg, 20% yield).

#### Oleanoyl vanillamide (**2b**):

white powder; IR ν_max_ (KBr) 3544, 3465, 3158, 1770, 1653, 1515, 1455, 1379, 1235, 1205, 1034, 854, 816, 739, cm^–1^; ^1^H NMR (500 MHz, CDCl_3_) δ 6.76 (1H, d, *J* = 8.0 Hz, H-5′), 6.73 (1H, d, *J* = 2.0 Hz, H-2’), 6.68 (1H, dd, *J* = 8.0, 2.0 Hz, H-6’), 5.24 (1H, t, *J* = 3.6 Hz, H-12), 4.24 (2H, s, H-7’), 3.81 (3H, s, H-8’), 3.13 (1H, dd, *J* = 10.7, 5.3 Hz, H-3), 2.81 (1H, dd, *J* = 14.0, 4.6 Hz, H-18), 2.00 (1H, td, *J* = 14.7, 5.4 Hz, H16-a), 1.82 (1H, m, H-11), 1.81 (1H, m, H-22a), 1.70 (1H, m, H-15a), 1.68 (1H, m, H-16b), 1.64 (1H, m, H-22b), 1.62 (1H, m, H-19a), 1.56 (1H, m, H-1a), 1.53 (2H, m, H-2), 1.48 (1H, m, H-6a), 1.47 (1H, m, H-9), 1.38 (1H, dd, *J* = 12.4, 3.3 Hz, H-7a), 1.31 (2H, m, H-6b and H-21a), 1.26 (1H, m, H-7b), 1.19 (1H, m, H-21b), 1.11 (1H, m, H-19b), 1.09 (3H, s, H-27), 1.07 (1H, m, H-15b), 0.91 (3H, s, H-23), 0.89 (1H, m, H-1b), 0.87 (3H, s, H-30), 0.85 (3H, s, H-29), 0.83 (3H, s, H-25), 0.71 (3H, s, H-24), 0.69 (3H, s, H-26), 0.66 (1H, dd, *J* = 11.7, 1.6 Hz, H-5); ^13^C NMR (CDCl_3_, 126 MHz) δ 175.6 (C, C-28), 147.1 (C, C-3′), 145.1 (C, C-4′), 143.0 (C, C-13), 129.6 (C, C-1′), 122.9 (CH, C-12), 120.5 (CH, C-6′), 114.7 (CH, C-5′), 110.9 (CH, C-2′), 78.8 (CH, C-3), 55.8 (CH_3_, C-8′), 55.2 (CH, C-5), 47.5 (CH, C-9), 46.8 (C, C-17), 45.7 (CH_2_, C-19), 43.4 (CH_2_, C-7′), 41.7 (C, C-14), 41.2 (CH, C-18), 39.3 (C, C-8), 38.7 (C, C-4), 38.5 (CH_2_, C-1), 37.0 (C, C-10), 33.7 (CH_2_, C-21), 32.9 (CH_3_, C-29), 32.7 (CH_2_, C-7), 32.4 (CH_2_, C-22), 30.6 (C, C-20), 28.0 (CH_3_, C-23), 27.7 (CH_2_, C-15), 26.7 (CH_2_, C-2), 25.6 (CH_2_, C-27), 23.4 (CH_3_, C-30), 23.4 (CH_2_, C-11), 23.0 (CH_2_, C-16), 18.3 (CH_2_, C-6), 16.8 (CH_3_, C-26), 15.6 (CH_3_, C-24), 15.3 (CH_3_, C-25); HR-ESIMS *m*/*z* 591.4273 [M + H]^+^ (calcd for C_38_H_57_NO_4_ 591.4288).

#### Ursoloyl vanillamide (**3b**):

white powder; IR ν_max_ (KBr) 3574, 3465, 3180, 1770, 1660, 1520, 1405, 1365, 1260, 1215, 1043, 859 cm^–1^; ^1^H NMR (500 MHz, CDCl_3_) δ 6.80 (1H, d, *J* = 2.0 Hz, H-2′), 6.69 (1H, d, *J* = 8.0 Hz, H-5′), 6.63 (1H, dd, *J* = 8.0, 1.9 Hz, H-6′), 5.18 (1H, t, *J* = 3.8 Hz, H-12), 4.14 (2H, d, *J* = 5.8 Hz, C-7′), 3.74 (3H, s, H-8′), 3.00 (1H, dt, *J* = 10.6, 5.3 Hz, H-3), 2.16 (1H, d, *J* = 11.2 Hz, H-18), 2.07 (2H, td, *J* = 13.4, 4.1 Hz, H-16a), 1.84 (2H, m, H-11), 1.80 (1H, m, H-15a), 1.70 (2H, m, H-22), 1.63 (1H, bd, *J* = 13.5 Hz, H-16b), 1.53 (1H, dd, *J* = 12.7, 3.4 Hz, H-1a), 1.46 (5H, m, H-9, 21-a, 6-a, H-7a, H-2), 1.37 (1H, m, H-19), 1.30 (1H, m, H-6b, H-7b), 1.25 (1H, m, H-21b), 1.04 (3H, s, H-27), 1.04 (1H, m, H-15b), 0.95 (1H, m, H-20), 0.92 (3H, d, *J* = 5.6 Hz, H-29), 0.90 (3H, s, H-23), 0.90 (1H, m, H-1b), 0.87 (3H, s, H-25), 0.82 (3H, d, *J* = 6.3 Hz, H-30), 0.73 (3H, s, H-26), 0.68 (3H, s, H-24), 0.67 (1H, m, H-5). ^13^C NMR (DMSO-*d*6, 126 MHz) δ 174.4 (C, C-28), 147.4 (C, C-3′), 145.3 (C, C-4′), 137.4 (C, C-13), 130.2 (C, C-1′), 125.3 (CH, C-12), 119.6 (CH, C-6′), 115.1 (CH, C-5′), 111.6 (CH, C-2′), 76.8 (CH, C-3), 55.5 (CH_3_, C-8′), 54.8 (CH, C-5), 52.3 (CH, C-18), 47.5 (C, C-17), 47.0 (CH, C-9), 41.9 (CH_2_, C-7′), 41.7 (C, C-14), 40.0 (C, C-8), 39.1 (C, C-4), 38.4 (CH_2_, C-1), 38.3 (C, C-10), 38.3 (2 CH, C-20 and C-19), 36.5 (CH_2_, C-22), 32.7 (CH_2_, C-21), 30.0 (CH_2_, C-7), 28.2 (CH_3_, C-23), 27.5 (CH_2_, C-15), 27.0 (CH_2_, C-2), 23.8 (CH_2_, C-16), 23.1 (CH_3_, C-27), 22.9 (CH_2_, C-11), 20.9 (CH_3_, C-29), 18.0 (CH_2_, C-6), 17.1 (CH_3_, C-26), 16.9 (CH_3_, C-30), 16.1 (CH_3_, C-24), 15.3 (CH_3_, C-25); HR-ESIMS *m*/*z* 591.4293 [M + H]^+^ (calcd for C_38_H_57_NO_4_ 591.4288).

### Molecular Docking and Molecular Dynamics Studies

The starting ligand geometry of the ligands was built with Ghemical 2.99^18^ and energy minimized at molecular mechanics level first, using Tripos 5.2 force field parametrization^19^ and then optimized using the GAMESS program^20^ at the Hartree–Fock level with the STO-3G basis set, followed by a single-point HF energy evaluation at the 6-31G* level to derive the partial atomic charges for the ligand by the RESP procedure.^21^ Docking studies were performed with AutoDock 4.2.^22^ hTRPV1 (PDB id: 5IRX) and the ligands were processed with AutoDock Tools (ADT) package version 1.5.6rc1 to merge nonpolar hydrogens, calculate Gasteiger charges, and select rotatable side chain bonds. Grid dimensions of 60 × 50 × 60, respectively, centered in the binding pocket, were generated with the program AutoGrid 4.2 included in the Autodock 4.2 distribution, with a spacing of 0.375 Å. A total of 100 molecular docking runs for each docking calculation were performed adopting a Lamarckian Genetic Algorithm (LGA) and the protocol already published.^23^ Flexibility was used for all rotatable bonds of the docked ligands. For each docking run, the best not-redundant poses in terms of binding energy values were selected as representatives and underwent energy minimization with the Amber16 package^24^ using the ff14SB version of AMBER ff14SB force field for the protein and gaff parameters for the ligand. UCSF Chimera 1.14^25^ was used for figures of the molecular complexes. The energy-minimized complexes were embedded in a POPC bilayer using the charmmgui web-interface, and then MD simulations in the membrane environment were carried out with the pmemd.cuda module of the Amber16 package, using lipid 14 (lipids), ff14SB force (protein), and gaff (ligand) force field parametrization. MD production runs were carried out for 100 ns. The Cpptraj module of AmberTools16 was used for trajectory analysis. The full MD protocol has been published elsewhere.^27^

### TRPV1 Channel Assay

Compound effects on intracellular Ca^2+^ concentration ([Ca^2+^]_i_) were determined using the selective intracellular fluorescent probe for Ca^2+^ Fluo-4, and assays were performed as described.^26^ Briefly, HEK-293 cells, stably transfected with recombinant human TRPV1 (selected by Geneticin 600 μg mL^–1^) or not transfected were cultured in EMEM + 2 mM glutamine +1% nonessential amino acids + 10% FBS and maintained at 37 °C with 5% CO_2_. The day of the experiment the cells were loaded in the dark at rt for 1 h with Fluo-4 AM (4 μM in DMSO containing 0.02% Pluronic F-127). After that, the cells were rinsed and resuspended in Tyrode’s solution (145 mM NaCl, 2.5 mM KCl, 1.5 mM CaCl_2_, 1.2 mM MgCl_2_, 10 mM d-glucose, and 10 mM HEPES, pH 7.4), then transferred to a quartz cuvette of a spectrofluorimeter (PerkinElmer LS50B; λ_EX_ = 488 nm, λ_EM_ = 516 nm) under continuous stirring. Cell fluorescence before and after the addition of various concentrations of test compounds was measured normalizing the effects against the response to ionomycin (4 μM). The potency of the compounds (EC_50_ values) is determined as the concentration required to produce half-maximal increases in [Ca^2+^]_i_. Antagonist behavior is evaluated against the agonist of the TRPV1 capsaicin (100 nM) and analyzed by adding the compounds directly in the quartz cuvette 5 min before stimulation of cells with the agonist. IC_50_ is expressed as the concentration exerting a half-maximal inhibition of agonist effect, taking as 100% the effect on [Ca^2+^]_i_ exerted by capsaicin (100 nM) alone. Dose–response curve fitting (sigmoidal dose–response variable slope) and parameter estimation were performed with Graph-Pad Prism8 (GraphPad Software Inc.). All determinations were performed at least in triplicate.

## Supporting Information Available

^1^H and ^13^C NMR spectra of oleoylvanillamide (**2b**) and Figure S1 (best docking poses of **2a** and **3a**) and Figure S2 (distances between the centers of mass of methyl C-29 and monomers A and B) (PDF)
