# Supplementary material for: Discovery of a Remarkable Methyl Shift Effect in the Vanilloid Activity of Triterpene Amides
Source: J Nat Prod. 2020 Nov 2;83(11):3476–81. doi: 10.1021/acs.jnatprod.0c00639 (PMC8016363; doi:10.1021/acs.jnatprod.0c00639)
Supplement: Supplementary file 1 — np0c00639_si_001.pdf [file np0c00639_si_001.pdf]

SUPPORTING INFORMATION

Discovery of a Remarkable Methyl Shift Effect in the Vanilloid Activity of Triterpene Amides

Rosa Maria Vitale,† Cristina Avonto,‡ Danilo Del Prete,§ Aniello Schiano Moriello,^, ∥Pietro Amodeo,†

Giovanni Appendino,*,§ and Luciano De Petrocellis*,^

†Institute of Biomolecular Chemistry, National Research Council (ICB-CNR), Via Campi Flegrei 34, 80078 Pozzuoli (NA), Italy

‡National Center for Natural Products Research, Research Institute of Pharmaceutical Science, School of Pharmacy, The University of Mississippi, University, Mississippi 38677, United States

§Dipartimento di Scienze del Farmaco, Largo Donegani 2, 28100 Novara, Italy

^ Endocannabinoid Research Group (ERG), Institute of Biomolecular Chemistry, National Research Council (ICB-CNR), Via Campi Flegrei 34, 80078 Pozzuoli (NA), Italy

∥Epitech Group SpA, Saccolongo, Padova, Italy

^{1}H NMR Spectrum of oleanoyl vanillate (**2b**)……………………………………………………Page 2

^{13}C NMR Spectrum of oleanoyl vanillate (**2b**)……………………………………………………Page 3

Figure S1. Best docking poses of **2a** and **3a** ………….…………………………………………..Page 4

Figure S2: Distances between the centers of mass of methyl C29 and monomers A and B…...Page 5

Figure S3: Concentration-response curve of **2a** and **3a** on intracellular Ca^{2+} elevation in HEK-293 cells over-expressing human TRPV1…………………………………………………………..Page 6

Figure S4. Concentration-response curve of **2a** and **3a** on intracellular Ca^{2+} elevation in HEK-293 cells over-expressing human TRPV1 against 100nM of capsaicin (desensitization)………… Page 6

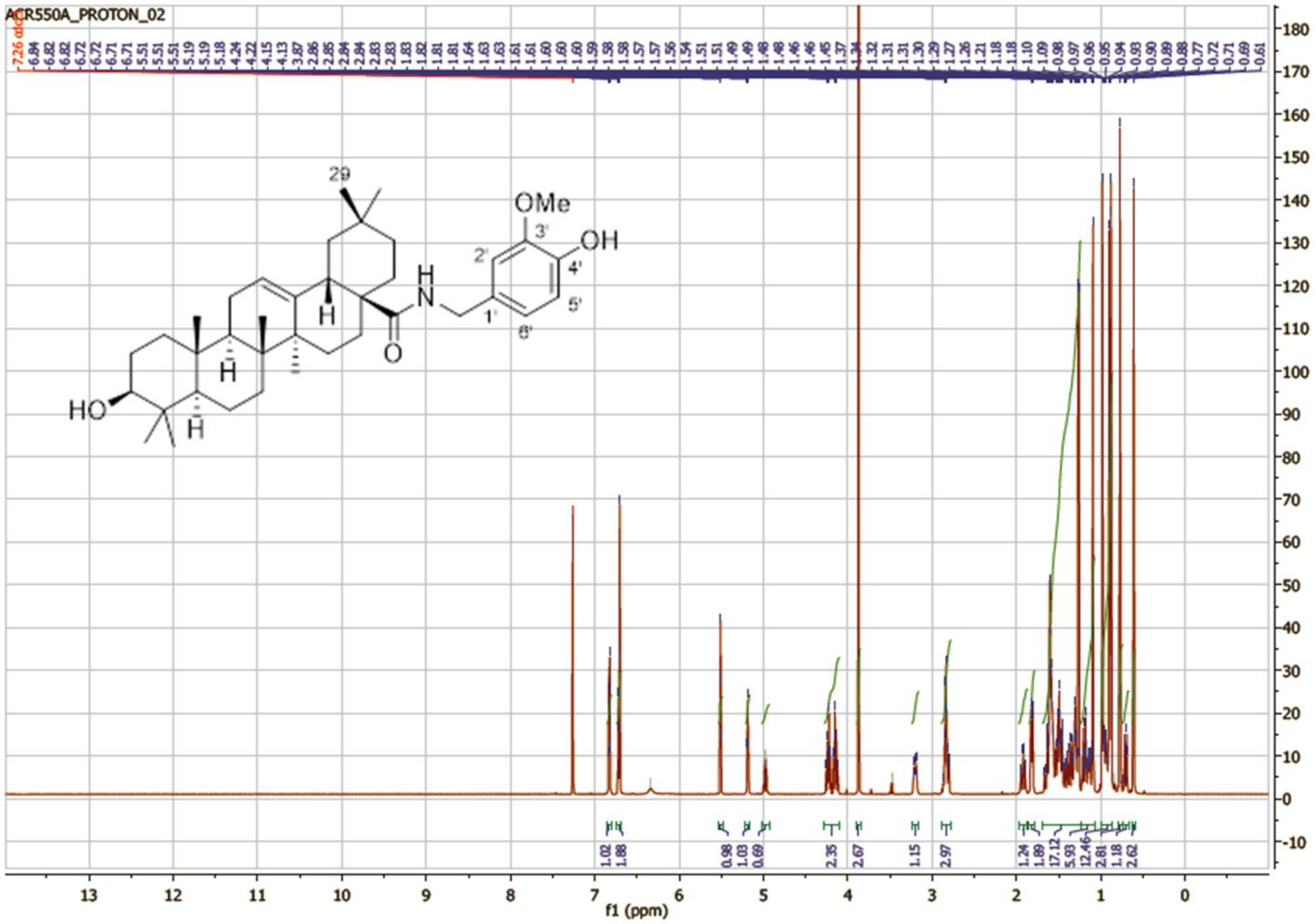

^{1}H NMR Spectrum of oleanoyl vanillate (**2b**) (500 MHz, $CDCl_3$).

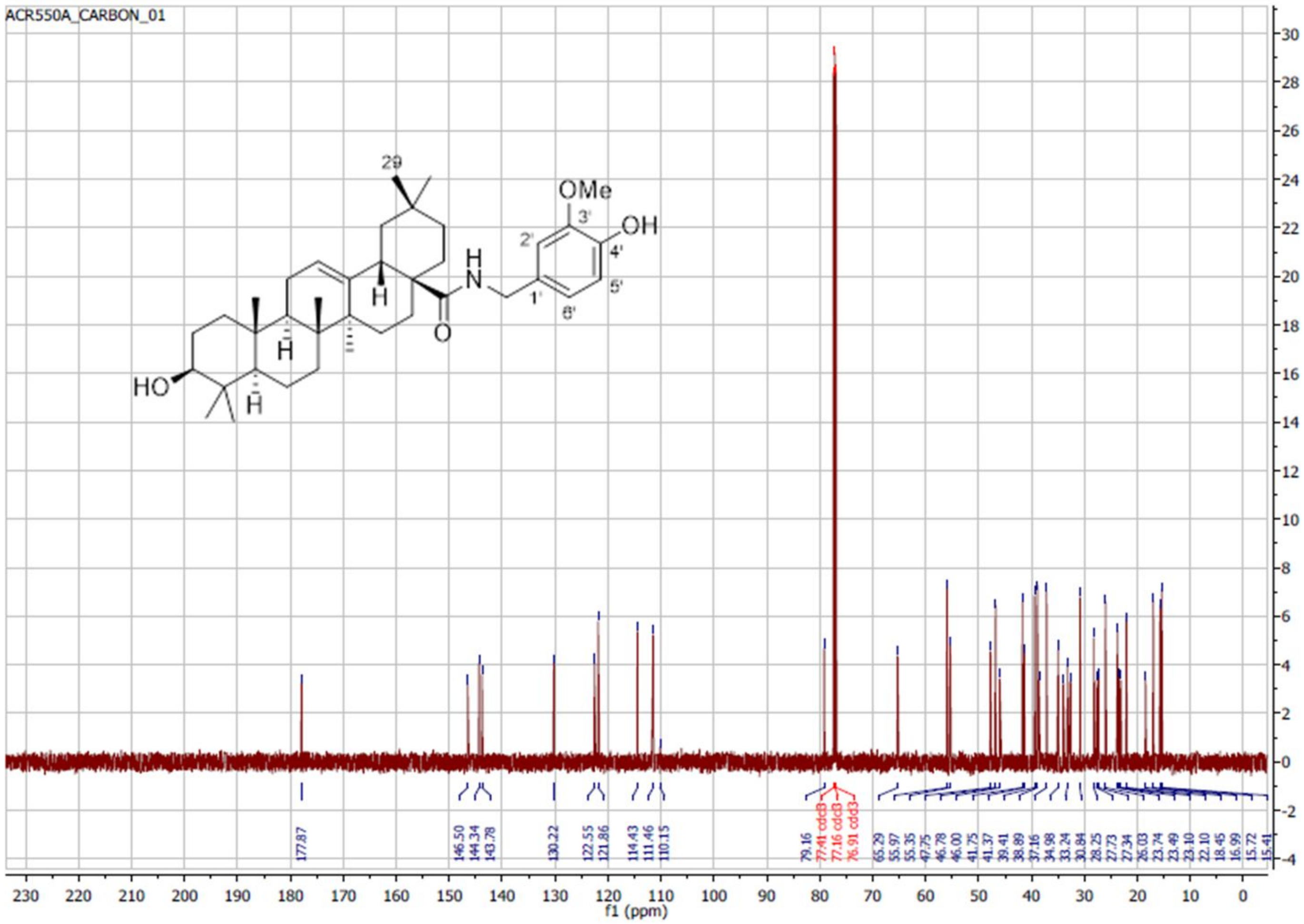

^{13}C NMR Spectrum of oleanoyl vanillate (**2b**) (126 MHz, $CDCl_3$).

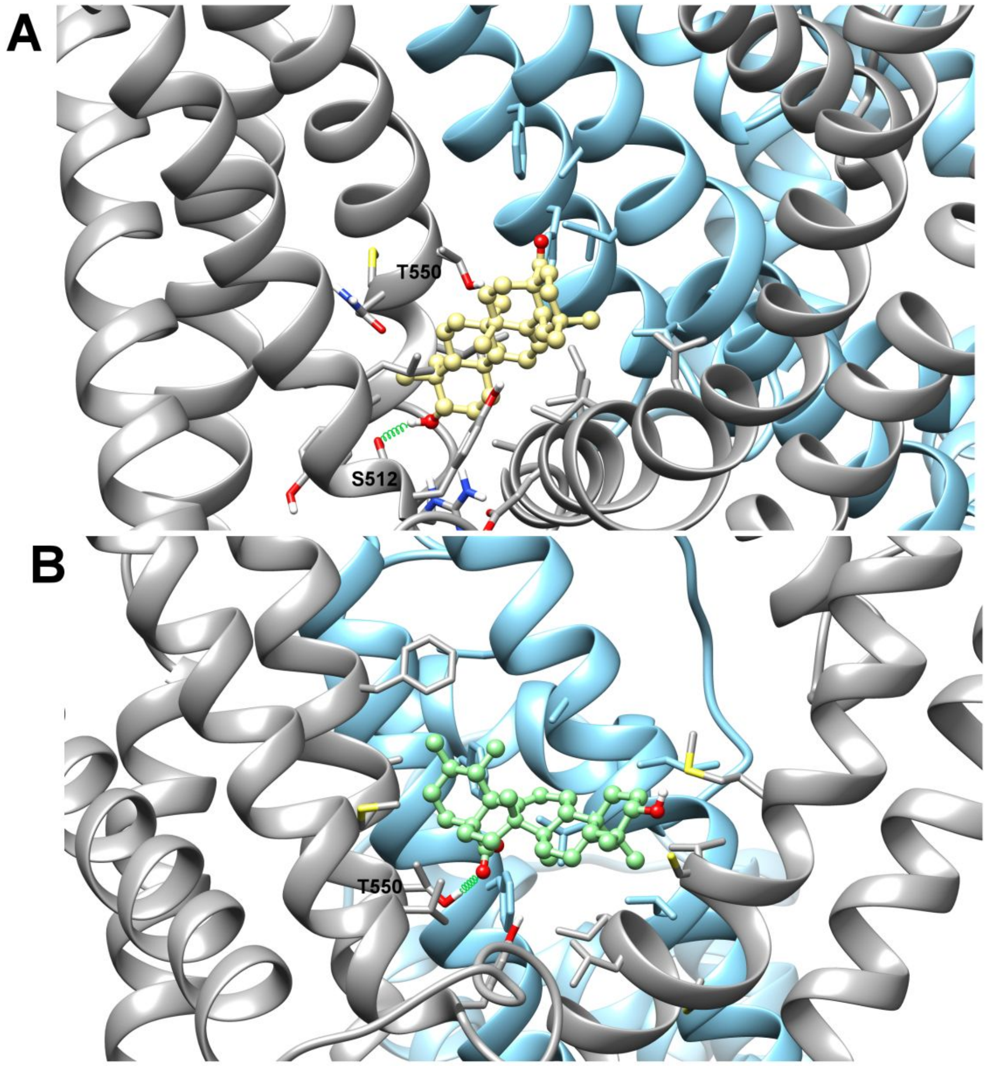

Figure S1. Best docking poses of compound **2a** (oleanolic acid, Panel A) and **3a** (ursolic acid, Panel B). Compounds **2a** and **3a** are represented in ball&stick and colored in kaki and light green, respectively. The H-bond is shown as green spring. TRPV1 monomers A and B are colored in sky blue and dark gray, respectively. Residues within 5Å from the ligand are shown in stick representation. Oxygen, nitrogen and sulfur atoms are colored in red, blue and yellow, respectively. Only polar hydrogens are shown and colored white.

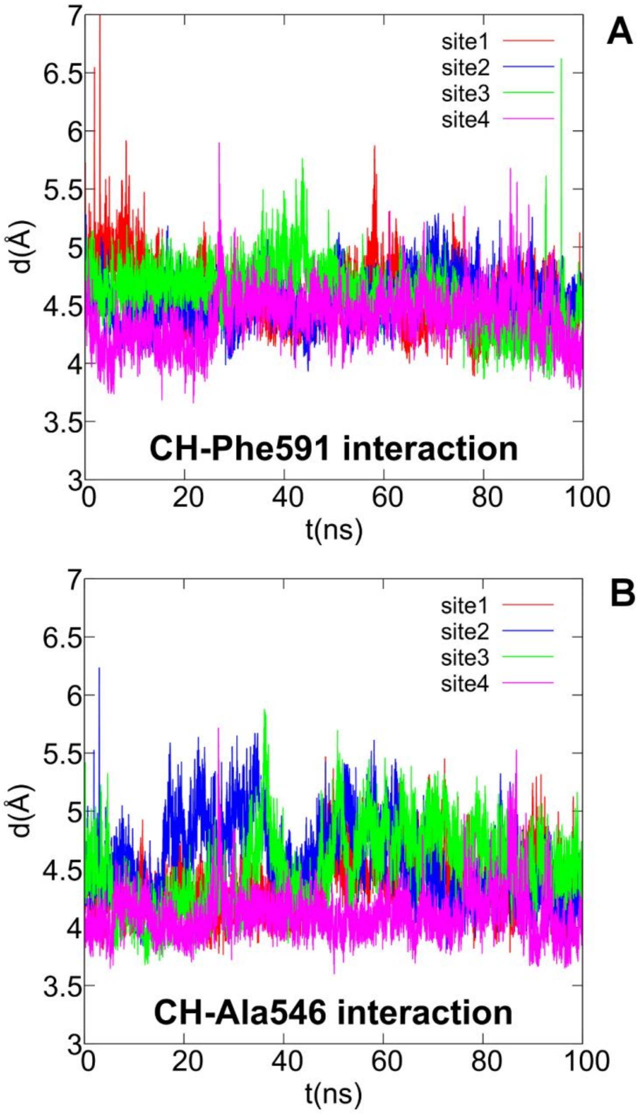

Figure S2. Distances between the centers of mass of methyl C29 and either monomer A Phe591 (A), or monomer B Ala549 (B) TRPV1 sidechains, over 100 ns of molecular dynamics. The plot lines were smoothed with an eleven-point window running average.

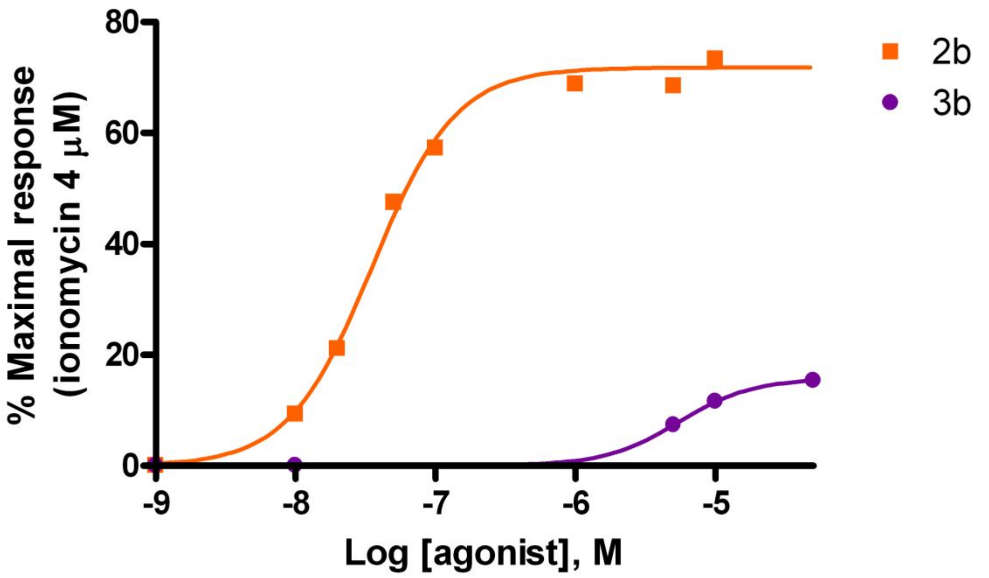

Figure S3. Effects of **2b** and **3b** on intracellular Ca^{2+} elevation in HEK-293 cells over-expressing human TRPV1. Data are expressed as % of the effect observed with 4 μM ionomycin.

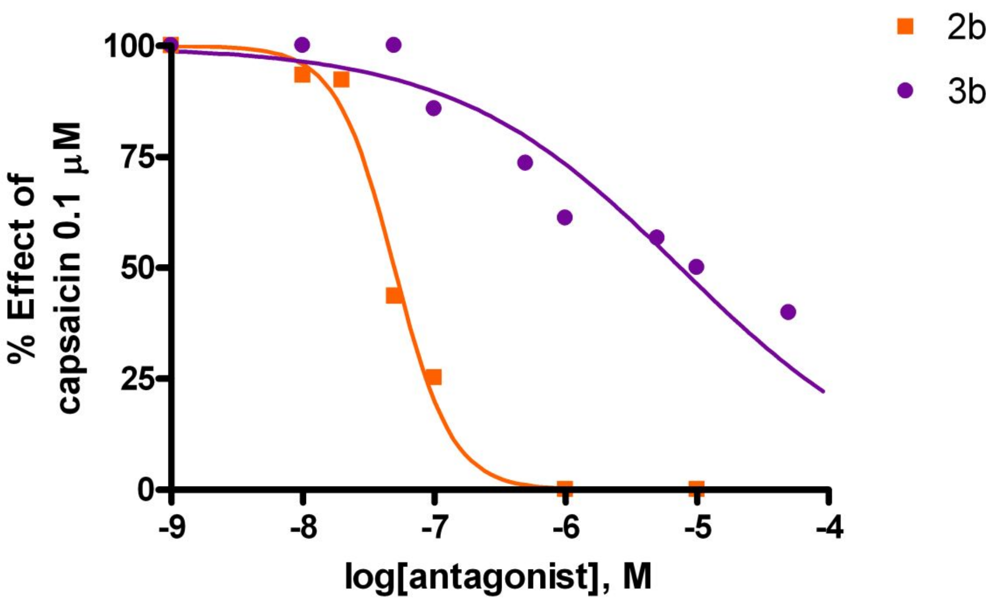

Figure S4. Desensitization by 5 min pre-incubation with **2b** and **3b** to the response to 100 nM capsaicin in HEK-293 cells over-expressing the human TRPV1. The effect on $[Ca^{2+}]_i$ exerted by 100 nM capsaicin alone was considered as 100 %
